# The Fibroid Removal in Sterility Treatment ‘‘FIRST’’ Survey: A European Society of Gynecology Online Questionnaire

**DOI:** 10.3390/jcm15134986

**Published:** 2026-06-26

**Authors:** Angelos Daniilidis, Georgios Grigoriadis, Michelle Nisolle, Camil Castelo-Branco, Stefano Angioni, Uzeyir Kalkan, Vito Cela, Lubomir Mikulasek, George Pados

**Affiliations:** 1 1st University Department of Obstetrics and Gynecology, Papageorgiou General Hospital, 56429 Thessaloniki, Greece; angedan@hotmail.com; 2 Department of Obstetrics and Gynecology, Hospital CHR Liège, University of Liège, 4000 Liège, Belgium; michelle.nisolle@citadelle.be; 3 Clinical Institute of Gynecology, Obstetrics and Neonatology, Hospital Clinic de Barcelona, 08036 Barcelona, Spain; ccastelobranco@gmail.com; 4 Division of Gynecology and Obstetrics, Department of Surgical Sciences, University of Cagliari, 09042 Cagliari, Italy; sangioni@yahoo.it; 5 Department of Obstetrics and Gynecology, Koc University Hospital and School of Medicine, Istanbul 34010, Turkey; uzekal@hotmail.com; 6 Department of Obstetrics and Gynecology, University of Pisa, 56126 Pisa, Italy; celav2001@gmail.com; 7 Department of Obstetrics and Gynecology, Centre for Fertility Preserving Treatment of Uterine Fibroids, 10916 Živoninská, Prague, Czech Republic; mikulasekl@icloud.com; 8 Department of Obstetrics and Gynecology, The European Interbalkan Medical Center, 57001 Thessaloniki, Greece; padosgyn@gmail.com

**Keywords:** fibroids, myomectomy, infertility, laparoscopy, hysteroscopy

## Abstract

**Background/Objectives:** The clinical management of uterine fibroids in the context of infertility is characterized by significant heterogeneity. The aim of our study was to record the participants’ views and clinical practices regarding minimally invasive, fertility-sparing management of fibroids, focusing on fertility outcomes. **Methods:** An online survey was distributed to members of the European Society of Gynecology (ESG), using a questionnaire comprising 27 questions. Questions 1 to 5 related to the participants’ background, while questions 6 to 27 related to the clinical management of fibroids. **Results:** A total of 98 participants completed the survey, of whom 83% (*n* = 82) practiced in European countries and 43% (*n* = 42) had completed specialist training in minimally invasive gynecological surgery. For FIGO 0–II fibroids, hysteroscopic removal was recommended by 94% (*n* = 92) of participants, although only 27% (*n* = 26) would do so in all cases, irrespective of the size and submucosal proportion. Anti-adhesion agents were used at least occasionally after the hysteroscopic removal of FIGO 0–II fibroids by 51% (*n* = 50) of participants. A clinically significant fibroid size was recognized by 57% (*n* = 56) of participants for FIGO III fibroids and by 51% (*n* = 50) for FIGO IV fibroids. The opinion was almost evenly divided on whether the distance between an intramural, non-cavity-distorting fibroid and the junctional zone affected the decision for removal: 49% (*n* = 48) considered that it did not, whereas 51% (*n* = 50) considered that it did, citing variable cut-off values. Most participants favored minimal-access approaches over laparotomy, whereas the use of robot-assisted laparoscopy was limited. **Conclusions:** Our results confirm the significant variation in clinical practice associated with fibroid management and underline the need for standardized care, based on high-quality evidence.

## 1. Introduction

Fibroids are the most common benign tumors of the female genital tract, with a prevalence ranging from 4.5% to 68.6% during female reproductive years [1]. Also known as leiomyomas (or myomas), fibroids arise in the myometrium and are stimulated by estrogen and progesterone; therefore, they may progress following menarche and before menopause, whereas they are more likely to regress in the post-menopausal period [2]. Although the majority of patients with fibroids are asymptomatic [3], patients may also experience a variety of symptoms such as heavy menstrual bleeding (HMB), pelvic pain or pressure symptoms [4]. Certain fibroids may also negatively affect female fertility and increase the risk of recurrent pregnancy loss [5], possibly due to a variety of mechanisms such as increased uterine peristalsis, neuroendocrine actions of the myoma pseudocapsule, and altered gene expression involved in endometrial receptivity [6,7]. The size and location of the fibroid may influence its impact on fertility [7]. Fibroids are also responsible for a significant financial burden, with direct healthcare costs estimated to range between 4 and 10 billion dollars in the USA [8].

Fibroids may vary greatly in terms of their size, number and localization. Depending on their location in relation to the endometrium, myometrium and uterine serosa, fibroids are commonly subclassified from 0 to 8, according to the widely-used International Federation of Gynaecology and Obstetrics (FIGO) “leiomyoma subclassification system” [9,10]: fibroids 0–2 are ‘‘submucous’’ and fibroids 3–8 are ‘‘other’’ (intramural: 3 and 4, subserosal: 5–7, other: 8), while the classification also recognizes hybrid fibroids that impact both the serosa and the endometrium (two numbers listed, separated by a hyphen). Given the heterogeneity of the condition and associated clinical picture, a “one size fits all” approach for patients with fibroids would be unrealistic.

In recent years, we have witnessed an undeniable increase in the uptake of minimally invasive, uterus-sparing approaches for the management of fibroids [11]. Although evidence supports the role of myomectomy in improving myoma-related symptoms and quality of life [12], the evidence is less clear regarding the impact of fibroids per se, as well as their removal, on fertility outcomes [5,13]. Following this, considerable variations worldwide have been documented on fibroid management in the context of fertility [14,15,16], while a recent systematic review highlighted the urgent need for high-quality guidelines to guide clinical practice [17].

The aim of our study, an online survey using a 27-question questionnaire distributed to gynecologists who are members of the European Society of Gynecology (ESG), was to capture their views and clinical practices regarding minimally invasive, fertility-sparing management of fibroids, focusing on fertility outcomes.

## 2. Materials and Methods

This was an anonymous, cross-sectional, questionnaire-based survey. The initial questionnaire consisted of 30 questions that, following review by all authors, was reduced to 27 questions in the final form. The questionnaire was developed by the author panel on the basis of the existing literature and clinical expertise; the three items removed during this internal review were considered redundant or ambiguous, and the wording of several questions was refined to improve clarity. The instrument was reviewed and approved by the ESG board; however, it was not formally validated, nor was it pilot-tested prior to dissemination. An anonymous, online survey was subsequently designed on the platform ‘’SurveyPlanet’’ (Survey Planet, LLC, Marina Del Rey, CA, USA; app.surveyplanet.com), including those 27 questions, which was approved by the board members of the ESG. An invitation link was automatically created by the aforementioned platform and shared with members of the ESG via email. No technical restrictions (for example, limiting responses by device or IP address) were applied to prevent duplicate submissions; however, the invitation was circulated only to ESG members, and the anonymous, voluntary format provided no incentive for repeat completion. The surveys were electronically completed and the answers collected, while survey administrators had real-time access to the data. Institutional Review Board (IRB) approval was not required for this study because it consisted of an anonymous, voluntary survey of healthcare professionals concerning their clinical opinions and practice patterns and did not involve patients, clinical interventions, or the collection of any identifiable personal or patient data; under applicable institutional and national regulations, research of this nature does not require formal ethics committee review. Approval by the ESG board of directors was nonetheless sought and granted in advance of initiating the survey.

Questions 1 to 5 related to the participants’ background, aiming to capture their relevant training and surgical experience (Table 1). Questions 6 to 27 related to the clinical management of fibroids (Table 2). Answering all questions, except question 8, was mandatory in order to proceed with the next question and submit the answers. For all questions except question 16, only one answer was possible. For question 16, multiple answers were acceptable.

A sample size calculation in advance was not applicable for this study. The survey was closed by the survey administrators at 3 months following the initiation of the survey. Therefore, the size of our availability sample was merely based on the number of participants that responded to our invitation and answered all questions of our online survey. A statistical analysis was performed using simple statistics, namely the counts and percentages for each answer. As this was a descriptive survey, no inferential statistical testing was performed. Since the answers were anonymous, it was not possible to group answers per country or geographical region.

Generative AI was used during the preparation of this manuscript to assist with language editing and to help draft and structure portions of the text. All AI-assisted output was reviewed and edited by the authors, who take full responsibility for the content and integrity of the work.

## 3. Results

A total of 98 participants completed our online survey. This corresponded to a response rate of 19.6%, based on the 500 invitations distributed to ESG members via the society’s mailing list. The majority (83%) of participants resided in Europe. The answers to questions 1 to 5 (with numbers and percentages per answer) are shown in Table 3, while the answers for questions 6 to 27 are shown in Table 4.

Among the 98 respondents, most were experienced clinicians, with 76% (*n* = 74) reporting more than 10 years of post-training experience; however, only 43% (*n* = 42) held a specialist qualification in minimally invasive gynecological surgery, and 33% (*n* = 32) performed fewer than 10 laparoscopic or robot-assisted myomectomies per year. For FIGO 0–II fibroids, hysteroscopic removal was broadly endorsed (94%, *n* = 92), yet the practice diverged with respect to the selection criteria: 27% (*n* = 26) would remove all submucosal fibroids, whereas 55% (*n* = 54) individualized the decision according to the fibroid size and submucosal proportion. Anti-adhesion measures after hysteroscopic myomectomy were used at least occasionally by 51% (*n* = 50) of participants, and 35% (*n* = 34) never performed second-look hysteroscopy.

For non-cavity-distorting FIGO III and IV fibroids, the opinion was markedly heterogeneous. A specific size threshold was regarded as clinically significant by 57% (*n* = 56) of participants for FIGO III fibroids and by 51% (*n* = 50) for FIGO IV fibroids, while 37% (*n* = 36) would not routinely recommend removal in couples trying to conceive. Conventional laparoscopy was the preferred surgical route (FIGO III, 41%, *n* = 40; FIGO IV, 63%, *n* = 62), whereas robot-assisted laparoscopy was selected by only 4% (*n* = 4), and almost four-fifths of participants (78%, *n* = 76) had never used a robotic approach. The distance of an intramural fibroid from the junctional zone influenced the decision for removal in 51% (*n* = 50) of participants, but not in the remaining 49% (*n* = 48). Barbed sutures were used at least occasionally by 71% of the respondents, and a three-month course of a GnRH analogue was the most frequently selected pre-treatment duration (FIGO III, 62%, *n* = 60).

**Figure 1:**
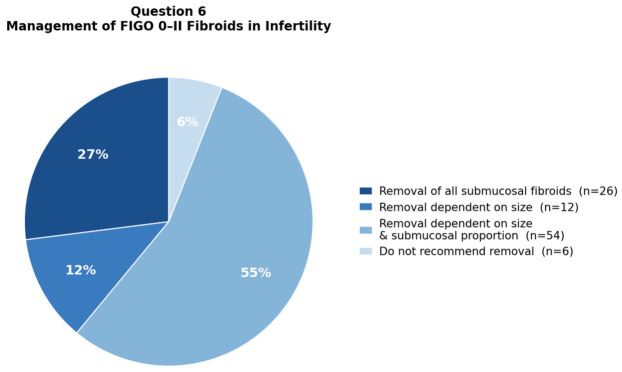
Pie chart for question 6: preferred management of FIGO 0–II fibroids in infertile patients. Remove all submucosal fibroids, 27% (*n* = 26); removal dependent on size, 12% (*n* = 12); removal dependent on size and submucosal proportion, 55% (*n* = 54); do not recommend hysteroscopic removal, 6% (*n* = 6).

**Figure 2:**
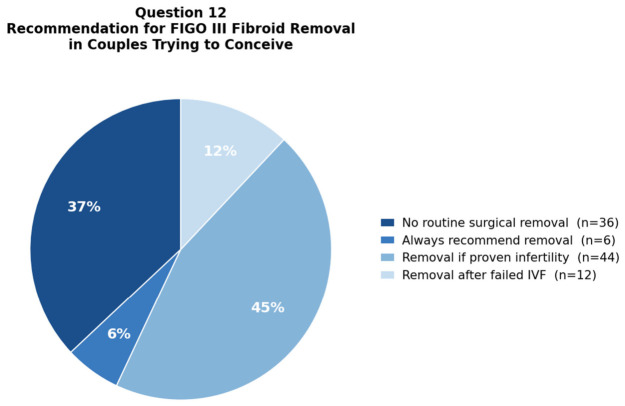
Pie chart for question 12: recommendation for surgical removal of FIGO III fibroids in couples trying to conceive. No routine removal, 37% (*n* = 36); always recommend, 6% (*n* = 6); removal if proven infertility, 45% (*n* = 44); removal after failed IVF, 12% (*n* = 12).

**Figure 3:**
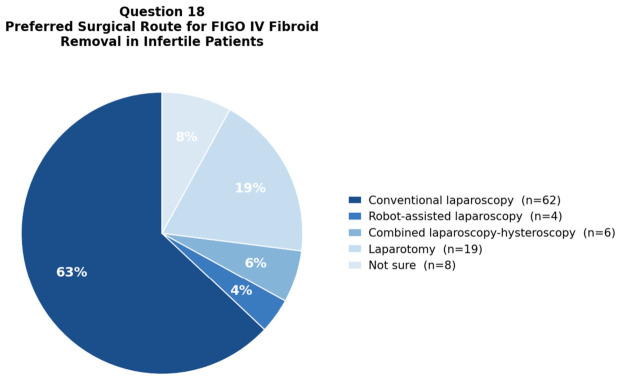
Pie chart for question 18: most commonly employed surgical route for FIGO IV fibroid removal in infertile patients. Conventional laparoscopy, 63% (*n* = 62); robot-assisted laparoscopy, 4% (*n* = 4); combined laparoscopy–hysteroscopy, 6% (*n* = 6); laparotomy, 19% (*n* = 18); not sure, 8% (*n* = 8).

**Figure 4:**
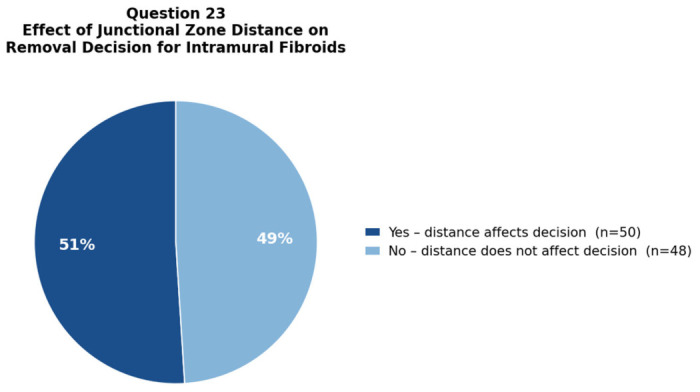
Pie chart for question 23: effect of junctional zone distance on decision for removal of intramural non-cavity-distorting fibroids. Yes, 51% (*n* = 50); no, 49% (*n* = 48).

Pie charts for question 6, question 12, question 18, and question 23 are demonstrated in Figure 1, Figure 2, Figure 3 and Figure 4, respectively.

## 4. Discussion

The results of our online survey among gynecologists, members of the ESG, reflect the paucity of high-quality evidence and lack of standardized care on many aspects of fertility-sparing management of fibroids. In line with this, a recent Cochrane review of four RCTs revealed uncertainty regarding the benefits of myomectomy over no intervention, in terms of clinical pregnancy rates and pregnancy loss risk reduction [5].

For FIGO 0–II fibroids, the majority of participants recommended hysteroscopic removal in infertile patients (94%); however, only 27% would remove them in all cases, regardless of the size and proportion of submucosal component. In contrast, 55% (*n* = 54) of participants reserved removal for fibroids selected according to both their size and submucosal proportion. Submucous fibroids present a number of unique features compared to outer myometrium fibroids: firstly, they are likely to originate in the junctional zone (JZ) of the myometrium [14]. Furthermore, submucous fibroids appear to have higher numbers of estrogen and progesterone receptors [18], as well as fewer karyotype aberrations compared to outer myometrium fibroids [19], which may explain why they are less likely to exhibit significant increases in size over time. Submucous fibroids are believed to have a negative effect on fertility through a variety of possible mechanisms, such as anatomical distortion of the uterine cavity [20], impaired junctional zone peristalsis [21], altering levels of inflammation [22], and molecular changes at the level of the endometrium [23]. A systematic review demonstrated that patients with submucous myomas had significantly lower clinical pregnancy and implantation rates compared to infertile controls, while hysteroscopic removal of submucous fibroids led to improved clinical pregnancy rates (but not live birth rates) compared to submucous fibroids left in situ [24]. According to the American Society of Reproductive Medicine (ASRM) clinical guidance, the removal of cavity-distorting fibroids (submucous or intramural with submucous component) should be considered in order to improve pregnancy rates even in asymptomatic patients, albeit insufficient evidence of its impact on live birth rates [13]. The International Federation of Fertility Societies (IFFS) suggests that removal should be considered in patients presenting with infertility [25]. Although there is insufficient evidence that the removal of submucous fibroids reduces the chances of early pregnancy loss in infertile patients [13], it should be acknowledged that patients with recurrent pregnancy losses form a distinct clinical entity. A retrospective cohort study of patients with myoma and recurrent early pregnancy losses observed a reduction in pregnancy losses following laparoscopic myomectomy; however, the number of patients with pregnancy losses was low [26].

With regards to measures to reduce the risk of adhesion formation following hysteroscopic myomectomy, half of the participants never use such measures (49%). It should be noted that the current guidelines provide only limited recommendations on adhesion prophylaxis after hysteroscopic myomectomy, which may partly account for this variability. A meta-analysis identified no benefit of using gel barriers on clinical or live birth rates; however, it reduced the presence of intra-uterine adhesions at second-look hysteroscopy [27]. Again, significant heterogeneity was noted among the participants on which method should be followed to reduce the adhesion formation risk, reflecting the lack of high-quality evidence comparing the available methods [28]. Similarly, the responses varied on the role of second-look hysteroscopy, with 35% of the participants never performing it. The American Association of Gynecologic Laparoscopists (AAGL) practice guidelines on submucosal fibroids state that second-look hysteroscopy may be effective for post-operative adhesions and could reduce the long-term risk of adhesion formation, particularly in cases of multiple submucosal myomectomies [14].

Non-cavity-distorting FIGO III and IV fibroids pose a unique fertility management challenge to the clinician, due to the lack of high-quality evidence guiding practice [29]. When asked which fibroid size they considered clinically significant in fibroid-associated infertility (questions 10 and 11), 57% of the participants regarded a particular size threshold as significant for FIGO III fibroids and 51% for FIGO IV fibroids, with a range of cut-off values cited; conversely, 43% and 49%, respectively, regarded fibroid size as not relevant. It needs to be acknowledged, however, that FIGO III fibroids, although anatomically-speaking intramural, abut the JZ and, therefore, may impair mechanisms critical to implantation independent of cavity distortion, likely having a more profound impact on fertility [29]. The ASRM practice committee guideline (2017) states that there is insufficient evidence that a certain size, number or location of non-cavity-distorting fibroids is associated with a reduced likelihood of achieving pregnancy or an increased risk of early pregnancy loss [13]. Similarly, the IFFS standard 10 and Australasian CREI Consensus Expert Panel on Trial Evidence (ACCEPT) consensus statement suggest that the impact of multiple fibroids and variation in size is uncertain [25,30]. A total of 37% of the participants would not routinely remove FIGO III or IV fibroids in couples trying to conceive. The IFFS Standard 10 states that there is no convincing evidence supporting the routine removal of intramural fibroids and that the decision to treat should be individualized, taking into account a multitude of factors [25]. Similarly, according to the ACCEPT consensus statement, there is insufficient evidence to determine whether myomectomy for intramural fibroids improves fertility outcomes [30], while the ASRM states that myomectomy is generally not advised to improve pregnancy outcomes in asymptomatic, infertile patients with non-cavity-distorting myomas [13]. Considerable variation was noted regarding the fibroid size cut-off to trigger removal prior to IVF, with nearly one fourth of participants stating that the size of FIGO III/IV fibroids is not relevant; despite the results of a recent meta-analysis identifying that patients with intramural, non-cavity-distorting fibroids of 2–6 cm have lower live birth rates (LBRs) following IVF compared to controls without fibroids [31], whether removal pre-IVF is indicated remains a matter of debate among experts [32], reflecting the conflicting evidence [5,33].

The preferable modality to diagnose and map fibroids in infertile patients also varied significantly between the participants, likely reflecting variations in local availability and protocols. A total of 23% utilize hysteroscopy, while the IFFS standard 10 recommends hysteroscopy in all patients suspected of having a submucous fibroid and those whose cavity is obscured by the presence of an intramural fibroid [25]. The majority utilize conventional laparoscopic myomectomy for FIGO III and IV fibroids, in line with the well-established benefits of minimal-access surgery over laparotomy [34], although evidence suggesting superiority in terms of fertility outcomes is currently lacking [5].

The vast majority of the participants check the integrity of the uterine cavity intra-operatively, following removal of FIGO III fibroids. Taking into account that such fibroids abut the endometrial cavity and a considerable risk of breaching the cavity during surgery exists, leading to intra-uterine adhesion formation and potentially serious gynecological and obstetric sequelae [35], this largely precautionary approach may seem reasonable, although it is not currently underpinned by high-quality evidence.

When asked about the duration of GnRH-agonist/antagonist treatment before removal of FIGO III and IV fibroids, the most popular answer was “3 months”: relevant guidelines confirm the need for “short-term” use only; however, no guidance on the exact course duration is given [25,30]. Of note, half of the participants stated that the distance between the inner portion of the intramural, non-cavity-distorting fibroid and the JZ does not affect their decision for removal and the other half stated that it does, with variable cut-off values given. Although fibroids with a shorter distance to the JZ may be linked to reduced chances of clinical pregnancy in women undergoing assisted reproductive technology (ART), the available guidance does not recommend removal solely based on the distance from JZ, nor does it provide cut-off values. Regarding the time period required between myomectomy and attempting natural conception or IVF, most responded that their decision would depend on factors like the number, size and surgical route. Although relevant guidelines do not provide a single answer and individualization appears reasonable, a systematic review identified no relationship between the risk of uterine rupture and the period of time between myomectomy and pregnancy [36].

Barbed sutures were used at least occasionally by the majority of participants (71%) in laparoscopic or robotic myomectomy, with 26% using them most of the time or always. Although their use may be associated with less operative time and blood loss and ease of use, international society guidelines do not recommend their use over conventional sutures, as no evidence of superior fertility outcomes has been observed [37].

Almost eight out of 10 participants had never used the robotic approach for minimally invasive myomectomy, a figure likely secondary to the lack of availability and training in certain healthcare settings. No clear guidance exists suggesting that the robotic approach is preferable to conventional laparoscopy for myomectomy, although a number of potential advantages of the robotic approach have been described [38], without evidence to date of improved reproductive outcomes.

The pronounced heterogeneity observed across most domains of practice is likely to be multifactorial. It may reflect the scarcity of high-quality, fertility-specific evidence and the resulting absence of uniform, prescriptive society guidance, but also differences in national and regional guidelines, variable access to technology and surgical training, institutional policies, and differing case-mix and resource availability between practice settings. Ambiguity in the interpretation of individual survey items may additionally have contributed to the divergence seen for some questions, such as the almost even split regarding the influence of the junctional-zone distance. Taken together, these findings are best interpreted as a reflection of genuine clinical equipoise rather than as evidence favoring any single approach.

Our study provides a realistic snapshot of fibroid-related care with a focus on fertility. Including a diverse population of participants in terms of experience, training and country of practice is reflective of the actual heterogeneity in the assessment and management of fibroids internationally. At the same time, a number of limitations need to be acknowledged: firstly, the relatively small number of participants. Furthermore, only half of the participants had undergone specialist training and four in 10 performed more than 10 laparoscopic or robot-assisted myomectomies per annum, suggesting that our sample comprised mostly clinicians with at least limited experience in the surgical management of fibroids. Although fibroid management in many countries is undertaken by generalists, so that our sample may reflect everyday rather than expert practice, the limited surgical experience of many of the respondents nonetheless constrains the generalizability of our findings to high-volume specialist centers. Lastly, our survey was anonymous; therefore, we were unable to group answers according to the participants’ background, with practices likely differing significantly among different levels of expertise and healthcare settings. Several further limitations warrant emphasis. The response rate was low (19.6%), raising the possibility of non-response and selection bias, since clinicians with a particular interest in, or strong views on, fibroid management may have been more inclined to participate. Non-responders may differ systematically from responders in their experience, sub-specialization, and practice setting; the responding sample may therefore over-represent clinicians with a specific interest in fibroid surgery and under-represent routine, non-specialist practice. The low response rate consequently limits the representativeness of the sample and the extent to which the findings can be generalized to the wider population of clinicians managing fibroids in the fertility context, and the results are best interpreted as indicative of practice patterns among engaged ESG members rather than as population-level estimates. As the survey captured self-reported practice, it could not be cross-validated against actual clinical activity or patient outcomes and is subject to recall bias. In addition, because the questionnaire was not formally validated or pilot-tested, its content validity rests on expert review alone; individual items may have been interpreted differently by respondents, and the measurement properties of the instrument were not formally established. The anonymous design precluded subgroup or correlation analyses by country, practice setting, or level of experience, which would otherwise have helped to explain the observed variation. Finally, the survey addressed surgical (excisional) management only and did not consider non-excisional or ablative techniques, such as radiofrequency ablation, myolysis, or uterine artery embolization; for women who wish to preserve fertility, myomectomy remains the preferred approach, as the available evidence on fertility and reproductive outcomes following these alternative techniques is currently limited [39].

## 5. Conclusions

The results of our online survey among gynecologists from variable backgrounds, members of the ESG, demonstrate a significant heterogeneity of opinion regarding most aspects of the assessment and management of fibroids in the fertility context, reflecting the lack of high-quality evidence and relevant formal society guidance. In particular, adequately powered randomized controlled trials comparing myomectomy with expectant management for non-cavity-distorting intramural fibroids, together with prospective multicenter registries, are needed in order to guide uniform, evidence-based care for fibroids in the context of fertility and sterility.

## Figures and Tables

**Table 1:** Questions 1 to 5.

Question 1	Please clarify your professional status
Question 2	Please clarify your years of experience post-training/fellowship
Question 3	Have you completed a specialist training/certification in minimally invasive gynecological surgery?
Question 4	How many laparoscopic/robot-assisted myomectomies do you perform per annum (on average, in the last 5 years)?
Question 5	How many hysteroscopic myomectomies do you perform per annum (on average, in the last 5 years)?

**Table 2:** Questions 6 to 27.

Question 6:	In your clinical practice, which of the following principles do you follow in a patient with a FIGO 0–II fibroid and infertility?
Question 7:	In your clinical practice, if hysteroscopic removal of a FIGO 0–II fibroid is decided, do you routinely use anti-adhesion agents?
Question 8:	If answer is no for the previous question, go directly to question number 9. If answer is yes for the previous question, what type of anti-adhesion do you usually use?
Question 9:	In your clinical practice, in case of hysteroscopic removal of a fibroid, do you routinely perform second-look hysteroscopic assessment of the endometrial cavity to assess for intra-uterine adhesions?
Question 10:	What size of a FIGO III fibroid do you consider to be significant in patients with fibroid-associated infertility?
Question 11:	What size of a FIGO IV do you consider to be significant in patients with fibroid associated infertility?
Question 12:	In your clinical practice, would you recommend surgical removal of a FIGO III fibroid in couples trying to conceive?
Question 13:	In your clinical practice, would you recommend surgical removal of a FIGO IV fibroid in couples trying to conceive?
Question 14:	In your clinical practice, if you recommend surgical removal of a FIGO III fibroid in infertile patients planned to undergo IVF, what fibroid size do you use as a cut-off?
Question 15:	In your clinical practice, if you recommend surgical removal of a FIGO IV fibroid in infertile patients planned to undergo IVF, what fibroid size do you use as a cut-off?
Question 16:	What modalities do you usually use to diagnose and map fibroids in patients with infertility?
Question 17:	In your clinical practice, should you decide to remove a FIGO III fibroid in an infertile patient, what is your most commonly employed surgical route?
Question 18:	In your clinical practice, should you decide to remove a FIGO IV fibroid in an infertile patient, what is your most commonly employed surgical route?
Question 19:	In your clinical practice, should you decide to remove a FIGO III fibroid in an infertile patient, do you routinely check during surgery the integrity of the uterine cavity following fibroid removal?
Question 20:	In your clinical practice, should you decide to remove a FIGO 0–II fibroid, do you use pre-treatment with gonadotrophin-releasing hormone agonist/antagonist?
Question 21:	In your clinical practice, should you decide to remove a FIGO III fibroid, for how long would you use pre-treatment with gonadotrophin-releasing hormone agonist/antagonist before the operation?
Question 22:	In your clinical practice, should you decide to remove a FIGO IV fibroid, for how long would you use pre-treatment with gonadotrophin-releasing hormone agonist/antagonist before the operation?
Question 23:	Regarding intramural, non cavity-distorting fibroids in infertile patients, does the distance from the junctional zone affect your decision for the removal?
Question 24:	If the answer to the previous question is yes, what is the distance of the fibroid from the junctional zone that would affect your decision?
Question 25:	Following myomectomy in infertile patients, how long do you advise your patients to wait before attempting to conceive/undergoing IVF?
Question 26:	In your clinical practice, do you use barbed sutures following laparoscopic/robot-assisted myomectomy?
Question 27:	Do you use the robotic approach for myomectomies and, if so, what percentage of myomectomies are done via the robotic approach?

**Table 3:** List of questions 1 to 5 (1st column) with possible answers per question in each row (numbers and percentages per answer). Abbreviations: *n* = number; NA = not applicable. In question 4, 27% (*n* = 26) of respondents, and in question 5, 14% (*n* = 14), selected ‘not applicable’, reflecting participants who do not perform the corresponding procedure.

Question	Answer A	Answer B	Answer C	Answer D	Answer E	Answer F	Answer G/NA
Q1	Trainee gynecologist (*n* = 6, 6%)	Consultant in private practice (*n* = 30, 31%)	Consultant in public hospital (*n* = 34, 35%)	Academic (*n* = 18, 18%)	Retired gynecologist (*n* = 6, 6%)	Other (*n* = 4, 4%)	NA (*n* = 0)
Q2	<5 (*n* = 8, 8%)	5–10 (*n* = 16, 16%)	>10 (*n* = 74, 76%)				
Q3	No (*n* = 56, 57%)	Yes (*n* = 42, 43%)					
Q4	<10 (*n* = 32, 33%)	10–30 (*n* = 16, 16%)	30–50 (*n* = 10, 10%)	>50 (*n* = 14, 14%)	NA (*n* = 26, 27%)		
Q5	<10 (*n* = 26, 27%)	10–20 (*n* = 20, 20%)	20–30 (*n* = 6, 6%)	>30 (*n* = 32, 33%)	NA (*n* = 14, 14%)		

**Table 4:** List of questions 6 to 27 (1st column, in bold) with possible answers per question in each row (numbers and percentages per answer). Abbreviations: *n* = number; N/A = not applicable; IUD = intra-uterine device; cm = centimeters; 2D = 2-dimensional; 3D = 3-dimensional; TVU = transvaginal ultrasound; mm = millimeters. Question 8 was conditional and was answered only by participants who reported using anti-adhesion agents. Question 16 permitted multiple responses; the percentages shown are expressed as a proportion of the total number of responses rather than of respondents, and therefore do not represent the proportion of participants selecting each modality. In questions 10 and 11, the categories ‘all sizes’ (any size considered significant) and ‘size irrelevant’ (size not taken into account) are mutually distinct.

Question	Answer A	Answer B	Answer C	Answer D	Answer E	Answer F	Answer G
Q6	Hysteroscopic removal of all submucosal fibroids (*n* = 26, 27%)	Removal dependent on size (*n* = 12, 12%)	Removal dependent on size and submucosal proportion (*n* = 54, 55%)	Do not recommend hysteroscopic removal (*n* = 6, 6%)			
Q7	Yes, always (*n* = 12, 12%)	Yes, sometimes (*n* = 38, 39%)	No, never (*n* = 48, 49%)				
Q8 (54 answers)	Copper IUD (*n* = 0)	Progesterone-releasing IUD (*n* = 20, 37%)	Foley’s balloon (*n* = 2, 4%)	Anti-adhesion agents (*n* = 20, 37%)	Oral hormonal treatment (*n* = 12, 22%)		
Q9	Yes, always (*n* = 18, 18%)	No, never (*n* = 34, 35%)	Yes, selected cases by fibroid size (*n* = 34, 35%)	Yes, selected cases by fibroid site (*n* = 12, 12%)			
Q10	>1 cm (*n* = 2, 2%)	>2 cm (*n* = 2, 2%)	>3 cm (*n* = 26, 27%)	>4 cm (*n* = 10, 10%)	>5 cm (*n* = 6, 6%)	All sizes (*n* = 10, 10%)	Size irrelevant (*n* = 42, 43%)
Q11	>1 cm (*n* = 0)	>2 cm (*n* = 6, 6%)	>3 cm (*n* = 2, 2%)	>4 cm (*n* = 22, 23%)	>5 cm (*n* = 16, 16%)	All sizes (*n* = 4, 4%)	Size irrelevant (*n* = 48, 49%)
Q12	No routine removal (*n* = 36, 37%)	Always recommend (*n* = 6, 6%)	Removal if proven infertility (*n* = 44, 45%)	Removal after failed IVF (*n* = 12, 12%)			
Q13	No routine removal (*n* = 36, 37%)	Always recommend (*n* = 10, 10%)	Removal if proven infertility (*n* = 38, 39%)	Removal after failed IVF (*n* = 14, 14%)			
Q14	>1–2 cm (*n* = 16, 16%)	3–4 cm (*n* = 34, 35%)	>5 cm (*n* = 22, 22%)	Size not relevant (*n* = 26, 27%)			
Q15	>1–2 cm (*n* = 6, 6%)	3–4 cm (*n* = 34, 35%)	>5 cm (*n* = 34, 35%)	Size not relevant (*n* = 24, 24%)			
Q16	2D TVU (*n* = 28, 29%)	3D TVU (*n* = 21, 21%)	Hysterosonography (*n* = 9, 9%)	Hysteroscopy (*n* = 22, 23%)	MRI (*n* = 17, 17%)	Other (*n* = 1, 1%)	
Q17	Conventional laparoscopy (*n* = 40, 41%)	Robot-assisted laparoscopy (*n* = 4, 4%)	Combined laparoscopy-hysteroscopy (*n* = 26, 27%)	Laparotomy (*n* = 14, 14%)	Not sure (*n* = 14, 14%)		
Q18	Conventional laparoscopy (*n* = 62, 63%)	Robot-assisted laparoscopy (*n* = 4, 4%)	Combined laparoscopy-hysteroscopy (*n* = 6, 6%)	Laparotomy (*n* = 18, 19%)	Not sure (*n* = 8, 8%)		
Q19	Yes, always (*n* = 68, 70%)	No, never (*n* = 14, 14%)	Sometimes (*n* = 16, 16%)				
Q20	Always (*n* = 6, 6%)	Occasionally (*n* = 38, 39%)	Never (*n* = 28, 29%)	Depending on size (*n* = 26, 26%)			
Q21	1 month (*n* = 10, 10%)	2 months (*n* = 8, 8%)	3 months (*n* = 60, 62%)	4 months (*n* = 0)	5 months (*n* = 2, 2%)	6 months (*n* = 8, 8%)	Other (*n* = 10, 10%)
Q22	1 month (*n* = 8, 8%)	2 months (*n* = 10, 10%)	3 months (*n* = 58, 59%)	4 months (*n* = 0)	5 months (*n* = 2, 2%)	6 months (*n* = 6, 6%)	Other (*n* = 14, 15%)
Q23	Yes, always (*n* = 50, 51%)	No, never (*n* = 48, 49%)					
Q24 (73 answers)	Distance < 3 mm (*n* = 14, 19%)	Distance 3–5 mm (*n* = 14, 19%)	Distance 5–10 mm (*n* = 12, 17%)	Any distance 1–2 cm (*n* = 5, 7%)	N/A (*n* = 28, 38%)		
Q25	Up to 3 months (*n* = 12, 12%)	3–6 months (*n* = 24, 25%)	>6 months (*n* = 22, 22%)	Depends on size/number/route (*n* = 38, 39%)	Other (*n* = 2, 2%)		
Q26	No, never (*n* = 28, 29%)	Yes but rarely (*n* = 18, 18%)	Yes, sometimes (*n* = 26, 27%)	Yes, most of the times (*n* = 20, 20%)	Yes, always (*n* = 6, 6%)		
Q27	No, never (*n* = 76, 78%)	Yes, <1/3 (*n* = 16, 16%)	Yes, 1/3–2/3 (*n* = 2, 2%)	Yes, >2/3 (*n* = 4, 4%)			

## Data Availability

Data supporting the results of this study are available from the corresponding author upon reasonable request.

## Abbreviations

The following abbreviations are used in this manuscript:

FIGO	International Federation of Gynaecology and Obstetrics
ESG	European Society of Gynecology
IVF	In Vitro Fertilization
ASRM	American Society for Reproductive Medicine
AAGL	American Association of Gynecologic Laparoscopists
IFFS	International Federation of Fertility Societies
ACCEPT	Australasian CREI Consensus Expert Panel on Trial Evidence
HMB	Heavy Menstrual Bleeding
JZ	Junctional Zone
ART	Assisted Reproductive Technology
LBR	Live Birth Rate
GnRH	Gonadotrophin-Releasing Hormone
IUD	Intra-Uterine Device
MRI	Magnetic Resonance Imaging
TVU	Transvaginal Ultrasound
2D	2-Dimensional
3D	3-Dimensional
RCT	Randomized Controlled Trial
IRB	Institutional Review Board
